# Prevalence and clinical impact of cachexia in chronic illness in Europe, USA, and Japan: facts and numbers update 2016

**DOI:** 10.1002/jcsm.12167

**Published:** 2016-11-02

**Authors:** Stephan von Haehling, Markus S. Anker, Stefan D. Anker

**Affiliations:** ^1^ Innovative Clinical Trial, Department of Cardiology and PneumologyUniversity of Göttingen Medical SchoolGöttingenGermany; ^2^ Department of Cardiology, Charité Campus Benjamin Franklin (CBF)BerlinGermany

**Keywords:** Cachexia, Wasting, Prevalence, Epidemiology, Treatment

## Abstract

Cachexia is a serious clinical consequence of almost all chronic diseases when reaching advanced stages. Its prevalence ranges from 5–15% in end‐stage chronic heart failure to 50–80% in advanced malignant cancer. Cachexia is also frequently occurring in patients with chronic kidney disease, chronic obstructive pulmonary disease (COPD) or neurological diseases, and rheumatoid arthritis. Mortality rates of patients with cachexia range from 15–25% per year in severe COPD through 20–40% per year in patients with chronic heart failure or chronic kidney disease to 20–80% in cancer cachexia. In the industrialized world (North America, Europe, and Japan) where epidemiological data are to some degree available, the overall prevalence of cachexia (due to any disease and not necessarily associated with hospital admission) is growing with the growth of the chronic illness prevalence, and it currently affects around 0.5–1.0% of the population, i.e. around 6–12 million people. From this, one can estimate that 1.5–2 million deaths are occurring in patients with cachexia per year. It is also a very significant health problem in other parts of the globe, but epidemiological data are scarce. The multifactorial nature of cachexia is now much better understood, and particularly, the role of inflammatory mediators and the imbalance of anabolism and catabolism are considered important therapeutic targets. Several approaches to develop cachexia and muscle wasting treatments have failed to be successful in phase III clinical trials, but new approaches are in development. Given the high prevalence and very high mortality associated with cachexia, advances are urgently needed for patients worldwide.

Cachexia is a serious clinical consequence of almost all chronic diseases when reaching advanced stages. Its prevalence ranges from 5–15% in end‐stage chronic heart failure, and it forms part of the terminal course of many patients with chronic kidney disease (CKD), chronic obstructive pulmonary disease (COPD), and rheumatoid arthritis. Also in many infections, cachexia can develop. Cachexia is associated particularly with cancer where the prevalence can reach 50–80% in advanced malignant cancer.

Using data from the Nationwide Inpatient Sample, Arthur *et al*. recently estimated the annual prevalence of cachexia‐attributed hospital admissions to community hospitals in the USA at over 160 000 cases.^1^ The median duration of stay was 6 days, compared with 3 days for non‐cachexia admissions. The median cost per hospitalization was more than $US10 000 per case of cachexia ($US4000 more than in non‐cachectic patients). Cachexia patients also experienced greater loss of function than those admitted with other diagnoses.^1^, ^2^

This provides only a partial view on the burden that cachexia condition imposes both on patients and on healthcare systems. Argiles *et al*. estimated that cachexia affects 50–80% of cancer patients and accounts as cause of death for up to 20% of cancer deaths.^3^ It is estimated that death normally ensues when weight loss exceeds 30–40%.^1^ However, many other patients (perhaps 50%) die with cachexia, but not due to cachexia. *Table* 1 gives updated estimates of the prevalence of cachexia in various chronic illnesses focusing on Europe. Data for Japan and the USA can also be estimated based on these data, as we have no reason to believe that the epidemiology of cachexia is fundamentally different between these regions.

**Table 1:** Estimates for the prevalence of cachexia in several chronic illnesses in Europe, the USA, and Japan

	Prevalence in population (%)	Patients at risk (%)	Prevalence in patients at risk (%)	Patients in Europe	Patients in the USA	Patients in Japan	1‐Year mortality (%)
COPD	3.5	15	35	1 400 000	600 000	230 000	15–25
(moderate severity)							
CHF	2.0	80	10	1 200 000	510 000	200 000	20–40
(NYHA II‐IV)							
Cancer	0.5	90	30	1 000 000	430 000	170 000	20–80
			(all types)				
Rheumatoid arthritis	0.8	20	10	120 000	50 000	20 000	5
(severe form)			(cachexia)				
CKD	0.1	50	50	190 000	80 000	30 000	20

Population assumptions (2013/14): Europe—742 million, the USA—319 million, and Japan—127 million.

Using the data in *Table* 1 and the estimates for the size of the whole population in the USA (319 million), Europe (742 million), and Japan (127 million) (data from 2013–14), the overall prevalence of cachexia in the population (due to any disease and not necessarily associated with hospital admission)—which is growing with the growth of the chronic illness prevalence—can be estimated at around 0.5–1.0% of the population, i.e. around 6–12 million people.

Mortality rates of patients with cachexia range from 15–25% per year in COPD through 20–40% per year in chronic HF and CKD up to 80% per year in some advanced cancers, like pancreatic cancer or non‐small cell lung cancer. From this and using the prevalence information above, one can estimate that 1.5–2 million deaths are occurring in patients with cachexia per year.

It is also a very significant health problem in other parts of the globe, but epidemiological data are scarce. Likely, cachexia is a much more frequent cause of death in Africa than in North America, Europe, or Japan. Efforts to improve the information on cachexia epidemiology from Africa, Asia, and South America are very much needed.

One key issue is the diagnosis of cachexia. Elements common to the way cachexia is defined include weight loss, low body mass index, fatigue, metabolic derangement, and biochemical markers of systemic inflammation.^4^, ^5^ A key concept is that while malnutrition is reversible when adequate amounts of food to meet needs are provided, cachexia is not.^6^ However, cachexia is defined: involuntary weight loss in patients reaching the terminal phase of many chronic diseases is common and severe enough to constitute a public health problem.^7^ Cachexia amply meets all the necessary criteria: it is a major contributor to morbidity and mortality, to impaired quality of life and to healthcare costs.

A number of treatments have been suggested for cachexia; however, therapies for the underlying illness remain at the forefront, and no direct treatment of cachexia is available as yet. The multifactorial nature of cachexia and muscle wasting is now much better understood, and particularly the role of inflammatory mediators, and the imbalance of anabolism and catabolism are considered important therapeutic targets.^8^, ^9^ Several approaches to develop cachexia and muscle wasting treatments have failed to be successful in phase III clinical trials, but new approaches are in development.

The precise criteria employed to define cachexia by different research groups vary, and this can have major implications for its reported prevalence. We prefer to define cachexia as suggested by Evans *et al*.^10^ Wallengren *et al*. found recently that the proportion of palliative care cancer patients classified as being cachectic ranged from 12 to 85% depending on the definition used.^11^

Among patients with cachexia, or at risk of developing it, we urgently need treatments that will enhance muscle mass or at least slow its depletion,^12^, ^13^ maintain body weight, improve strength, enhance the capacity for independent functioning, reduce frailty,^14^ and prolong survival.^15^ Given the high prevalence and very high mortality associated with cachexia, advances are urgently needed for patients worldwide.

## Conflict of interest

S.D.A. reports fees for consultancy and/or speaking from Bayer, Helsinn, Novartis, Pfizer, Stealth Peptides. and Vifor. S.D.A. reports grants from Vifor and Abbott Vascular. S.v.H. reports fees for consultancy and/or speaking for Vifor, Pfizer, Chugai, and Novartis. M.S.A. reports no conflicts of interest.
